# A Method for the Intelligent Identification of the Operating Conditions of Deep-Sea Submersibles and Its Application

**DOI:** 10.3390/s25226904

**Published:** 2025-11-12

**Authors:** Yi Zhang, Zihao Li, Jialing Tang, Kaixun He

**Affiliations:** 1 National Deep Sea Center, Qingdao 266237, China; zy592@ndsc.org.cn (Y.Z.); tjl@ndsc.org.cn (J.T.); 2 College of Electrical Engineering and Automation, Shandong University of Science and Technology, Qingdao 266590, China; 202483080116@sdust.edu.cn

**Keywords:** graph neural network, submersible, data driven, K-nearest neighbor, condition identification

## Abstract

The precise identification of operating conditions is a critical prerequisite for ensuring the safety of manned deep-sea submersibles, a task complicated by extreme environments and tightly coupled subsystems. Traditional methods, which often overlook the latent correlations among monitoring variables, are frequently insufficient for this high-stakes application. To address these issues, this paper proposes a novel working condition identification method tailored to the challenges of the deep-sea environment, which leverages graph neural networks to explicitly model the relationships between sensor variables. The proposed framework first dynamically constructs a graph structure from multi-sensor snapshots using the K-Nearest Neighbor (K-NN) algorithm, where edges represent high similarity in the submersible’s operational state. Subsequently, a graph neural network is employed to learn from this relational data. Furthermore, a model update strategy is introduced to enable the adaptive recognition of new, emerging operational conditions. A case analysis using real operational data from a manned deep-sea submersible demonstrates that the proposed method significantly enhances identification accuracy. Moreover, interpretability analysis reveals that the model learns physically meaningful patterns consistent with the submersible’s engineering principles, enhancing its trustworthiness.

## 1. Introduction

Deep-sea manned submersibles, as critical equipment for exploring and developing deep-sea resources, necessitate paramount operational safety and reliability [1]. In the complex and high-pressure underwater environment, a multitude of sensors are relied upon to monitor the status of navigation, power, hydraulic, and other subsystems in real time, generating massive volumes of multi-source time-series data. This data provides an essential foundation for understanding the operational mechanisms of manned submersibles and enabling intelligent health management. The application of data-driven methods such as deep learning to operational condition identification is regarded as the core prerequisite for achieving anomaly detection, fault, and intelligent decision-making [2,3].

Accurate operational condition identification carries critical significance in two aspects. First, it serves as the basis for operational safety. The precise recognition of different conditions, such as diving, cruising, and operating, allows for appropriate operational parameters and safety thresholds to be set for the system, enabling timely detection of abnormal deviations from the baseline state. Second, it constitutes the cornerstone of intelligent maintenance. Only when the current operational condition is correctly identified can the corresponding health assessment and diagnosis strategies be activated, thereby avoiding false alarms and missed detections and enhancing the overall intelligence of the system [4].

However, traditional methods for operational condition identification exhibit notable limitations. Mainstream approaches, such as Support Vector Machines and Random Forests, have typically treated multi-sensor data as a flattened feature vector, neglecting the complex nonlinear couplings and physical correlations among variables. For instance, changes in the propulsion system’s power may induce responses in hydraulic pressure and temperature—structural relationships that cannot be effectively captured by conventional methods. As a result, the model’s capacity to represent the dynamic behavior of the system remains limited, and the identification accuracy has been insufficient to meet practical safety requirements [5]. Therefore, it is imperative to develop multi-source information fusion and system coupling modeling technologies oriented toward engineering applications, so that a high-precision and highly reliable operational condition identification system suited to real deep-sea environments can be established, thereby ensuring operational safety and intelligent maintenance throughout complex submersible missions.

Data-driven operational condition identification technology for industrial equipment has progressively evolved from traditional machine learning methods to advanced deep learning frameworks. Early research primarily relied on classical classification algorithms. For instance, Ye Zhao et al. employed a decision tree model to effectively classify different operational states based on the operating parameters of photovoltaic arrays [6]. To handle more complex process data, researchers introduced feature engineering techniques. For example, Yang C et al. combined Principal Component Analysis (PCA) with Support Vector Machine (SVM) to improve the accuracy of process state identification through data dimensionality reduction and feature selection [7]. Similarly, D. A. Asfani et al. utilized wavelet transform to extract key features from motor current signals and applied a Naive Bayes classifier to identify specific abnormal conditions [8]. These methods laid the foundation for data-driven identification; however, their performance heavily relied on handcrafted features, limiting their effectiveness and generalization when applied to complex systems with multivariable and strong coupling characteristics, such as manned submersibles.

To reduce dependence on prior knowledge, methods based on intrinsic data distribution and neighborhood relationships have gained attention. To address the limitations of traditional K-means algorithms in identifying abnormal operational modes, Yin Chunyong et al. incorporated information entropy to improve clustering accuracy and convergence speed [9]. Guoi J Y et al. observed that conventional KNN methods, when handling multimodal data, tend to obscure subtle state deviations due to disparities in intermodal dispersion. They proposed a KNN identification method integrated with probability density (PD), significantly enhancing the model’s sensitivity to minor state changes [10]. These studies reflect a shift in mindset from “feature engineering” to “mining data structures.” In particular, the application of kNN highlighted the importance of “neighborhood relationships” in feature space for state identification. Nevertheless, these methods essentially still measure sample similarity in Euclidean space and fail to fundamentally address the core challenge of explicitly modeling the intrinsic non-Euclidean relational structures among various sensors (variables) in deep-sea manned submersibles.

The rise in deep learning has brought a paradigm shift to the field of condition identification. As noted by Syahril Ramadhan Saufi et al. in their review, a key advantage of deep learning lies in its ability to automate feature learning through deep architectures [11]. Among these, Convolutional Neural Networks (CNNs) and Recurrent Neural Networks (RNNs) are the two most widely applied models. Li Z W et al. systematically elaborated on the powerful local feature extraction capability of CNNs [12]. Olivier Janssens et al. directly input the frequency spectra of raw vibration signals into a CNN, achieving end-to-end identification of rotating machinery operational states [13]. To capture temporal dependencies in data, models centered around RNN and its variants—Long Short-Term Memory (LSTM) and Gated Recurrent Unit (GRU)—have been successively proposed. Wang J G et al. utilized an RNN to model the dynamics of an underwater vehicle’s thruster and performed online condition monitoring by analyzing residuals between model outputs and actual data [14]. Lei J H et al. leveraged the ability of LSTM to automatically learn long-term dependencies from multivariate time series, constructing an efficient operational state identification framework for wind turbines [15]. Furthermore, researchers have explored more complex hybrid architectures. For example, Zhao R et al. combined local feature engineering with a bidirectional GRU network (LFGRU), achieving high accuracy in state evaluation tasks across multiple types of mechanical systems [16].

Despite the remarkable success of the aforementioned deep learning models in automatically extracting temporal features, they generally share a fundamental limitation: they typically treat multivariate time series as a set of parallel and independent channels, failing to explicitly model the complex coupling relationships among different sensor variables in the “spatial” dimension [17]. The monitoring network of a deep submersible is inherently a graph, where nodes represent sensors and edges represent physical or statistical dependencies among them [18]. The “channel independence” assumption of traditional deep learning methods overlooks this critical system topology, making it difficult for the models to effectively detect condition transitions that are reflected through subtle changes in multivariate relational patterns.

In summary, existing condition identification methods either rely on handcrafted features or excel at capturing temporal dependencies while neglecting spatial relationships among variables. Therefore, there is an urgent need for a new paradigm capable of simultaneously and automatically learning both temporal and spatial dependencies within the data. Graph Neural Networks (GNNs), particularly Graph Convolutional Networks (GCNs), provide an ideal framework to address this issue [19,20]. However, applying GCNs to deep submersible condition identification faces a prerequisite: the graph structure (i.e., the adjacency matrix) is typically unknown in practical applications [21]. To address this research gap, this paper proposes a deep integration of the K-Nearest Neighbor (KNN) algorithm with Graph Convolutional Networks (GCNs). We innovatively leverage KNN to dynamically construct neighborhood relationships between samples in the feature space based on the data itself, thereby adaptively generating the graph structure. GCN is then employed to perform information propagation and feature aggregation on this graph, enabling accurate, robust, and adaptive identification of operational conditions in deep-sea manned submersibles.

The identification of operational conditions for manned deep-sea submersibles presents a distinct and formidable challenge, stemming from a unique confluence of factors. First, they operate under extreme conditions, including immense hydrostatic pressure and unpredictable deep-sea topographies, which generate highly non-stationary and complex sensor signals. Second, as high-consequence assets with a human crew, the demand for near-perfect accuracy and reliability is paramount. Most critically, a submersible is a complex ‘system of systems’ where power, hydraulic, and navigation subsystems are intricately interconnected. The subtle cascading correlations between these subsystems are often the earliest indicators of a changing operational state.

This unique context exposes two core challenges that hinder the application of traditional identification methods: First, these models generally overlook the inherent inter-sensor coupling effects, making it difficult to effectively capture the critical system-level state information described above. Second, most existing methods are offline, static models that lack the adaptive learning and generalization capabilities required to handle new conditions emerging during actual operation. To address these challenges, the main contributions of this paper are summarized as follows:

- A novel framework integrating dynamic graph construction with graph convolutional learning is proposed to explicitly model inter-sensor coupling relationships. By adaptively building graph structures via K-NN and performing end-to-end feature learning with GCN, the coupling effects among sensors are effectively transformed into structured representations, thereby enhancing both the accuracy and robustness of the identification model.
- A dynamic adaptation strategy comprising data preprocessing and model updating is designed to mitigate strong noise and address unknown conditions. An outlier detection method based on moving windows and MAD is introduced to filter noise, while an online update mechanism enables the model to iteratively adapt to emerging conditions, ensuring long-term effectiveness in dynamic environments.
- The superiority of the proposed method is validated on a real deep submersible dataset under noisy and multi-condition scenarios, where it significantly outperforms baseline models in identification accuracy and adaptability. Moreover, model interpretability is enhanced through SHAP, which provides visual explanations of sensor contributions to condition classification, thereby increasing the transparency and credibility of the decision process.

## 2. Condition Identification Method Based on Graph Structure and K-NN

### 2.1. Problem Definition

For the task of identifying operating conditions of deep-sea manned submersibles, the core problem of this study can be formally defined as accurately, robustly, and interpretably identifying the operating states of the submersible system in an online manner, based on time-series data streams collected from multi-source sensors. Assuming there are *M* sensors in the submersible’s motion system, the multivariate observation data collected at time *t* can be denoted as $X_{i}\in R^{M}$. Thus, all data collected over a period of time can be represented as $X=\{X_{1},X_{2},\ldots ,X_{N}\}$, where *N* is the total number of samples, and each sample contains *M* features. The labels can be expressed as a vector of length *N*, denoted as $y=\{y_{1},y_{2},\ldots ,y_{N}\}$, where each element corresponds to the operating condition label of a sample, with a total of K distinct conditions. The entire dataset can thus be represented as D=(X,y). According to the input requirements of Graph Convolutional Neural Networks, the data format must be transformed into a graph structure G=(X,A,y). In this framework, each node $x_{i}$ represents a multi-sensor snapshot of the submersible’s operational state at a specific moment, with its corresponding feature vector composed of simultaneous readings from all monitored sensors (e.g., depth, pressure, and velocity). The matrix X thus contains the feature vectors for all nodes. The edge connections, encoded in the adjacency matrix A, represent a high degree of operational similarity between two snapshots and are dynamically constructed using the K-NN algorithm, as detailed in Section 2.3. Finally, the vector *y* contains the ground truth operational condition label for each node, derived from mission logs. The resulting graph provides a rich, relational representation of the submersible’s operational data.

The graph convolution-based operational condition identification method aims to determine the current equipment condition by processing the graph-structured data input into the model. This process involves a function with learnable parameters. The learned function maps the input graph data to probability values over different operational conditions, where a higher probability indicates a greater likelihood that the deep submersible is in that specific condition. The function corresponds to a Graph Convolutional Neural Network (GCN), which effectively captures complex inter-node relationships to achieve accurate identification of equipment operational conditions.

The loss function adopted for model training is the cross-entropy loss, which is calculated as follows:

(1) $$L\left(\omega \right)=-\sum_{i=1}^{N}\sum_{c=1}^{K}y_{ic}\log\left(h_{\omega }{\left(x_{i}\right)}_{c}\right)$$

**Figure 1:**
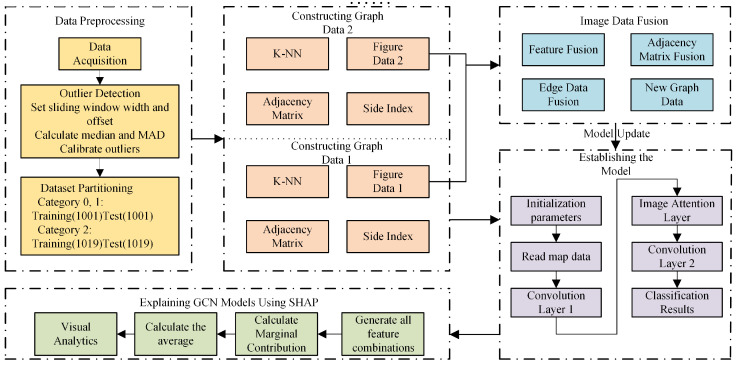
Flow chart of the whole algorithm.

In Equation (1), the total loss L(ω) is calculated over the entire dataset. Here, *N* represents the total number of multi-sensor snapshots collected from the submersible, and *c* is the number of distinct operational conditions (e.g., ‘descent’ and ‘cruising’). For a given snapshot $x_{i}$, $h_{\omega }{\left(x_{i}\right)}_{c}$ is the model’s predicted probability that the submersible is in the c-th operational condition. The term $y_{ic}$ represents the one-hot-encoded ground truth label derived from the submersible’s mission logs, where $y_{ic}=1$ if the true condition is *c*, and 0 otherwise. Minimizing this cross-entropy loss function is the objective of the model training process. A submersible operational condition identification method, enhanced by K-Nearest Neighbor graph convolutional training, is illustrated in the overall workflow in Figure 1.

### 2.2. Graph Convolutional Networks

Graph Convolutional Networks (GCNs) are deep learning models designed for graph-structured data, capable of capturing both local and global dependencies among nodes [22]. In the context of deep-sea submersible condition identification, multi-sensor data exhibits complex coupling relationships and nonlinear interactions [23]. By representing sensor variables and their interrelationships as a graph, GCNs can explicitly leverage physical or statistical associations among variables, thereby improving the identification of complex system states.

A graph is generally represented as G=(V,E), where *V* denotes the set of nodes and *E* the set of edges. This structure is mathematically described as:

(2) $$H^{\left(l\right)}=\sigma \left({\tilde{D}}^{-\frac{1}{2}}\tilde{A}{\tilde{D}}^{-\frac{1}{2}}H^{\left(l-1\right)}W^{\left(l\right)}\right)$$

Here, $H^{\left(l\right)}$ represents the matrix of node features at layer *l*, where each row is the feature vector of a specific sensor snapshot from the submersible. The adjacency matrix $\tilde{A}$ encodes the neighborhood structure of these snapshots, where an edge signifies high similarity in the submersible’s operational state. The GCN propagation rule thus models how the feature representation of one snapshot is updated by aggregating the features of other, highly similar snapshots. The matrix $W^{\left(l\right)}$ contains the learnable parameters that transform these aggregated features.The overall procedure of the graph convolutional neural network is summarized in Algorithm 1.
**Algorithm 1** Graph Convolutional Neural Network**Require:** Node feature matrix X, Normalized adjacency matrix A, Dropout rate *p*, Number of epochs *T*, Batch size *m*.**Ensure:** Final model parameters ω, Prediction matrix P.1:Initialize model parameters $\omega =\{\omega^{\left(0\right)},\omega^{\left(1\right)}\}$.2:**while** ω has not converged **do**3:    **for** epoch = 1 to *T* **do**4:        $H^{\left(1\right)}\leftarrow \mathrm{ReLU}\left(A\cdot X\cdot \omega^{\left(0\right)}\right)$5:        $Z\leftarrow A\cdot H^{\left(1\right)}\cdot \omega^{\left(1\right)}$6:        P←Softmax(Z)7:        $L\leftarrow -\sum_{i=1}^{N}\sum_{c=1}^{K}y_{ic}\log\left(h_{\omega }{\left(x_{i}\right)}_{c}\right)$8:        Compute gradients $\nabla \omega^{\left(0\right)},\nabla \omega^{\left(1\right)}$9:        Update parameters ω using Adam optimizer:         $\omega \leftarrow \mathrm{Adam}(\nabla_{\omega }\frac{1}{m}\sum_{i=1}^{m}L_{i},\omega ,\alpha )$10:    **end for**11:**end while**12:**return** ω,P

**Figure 2:**
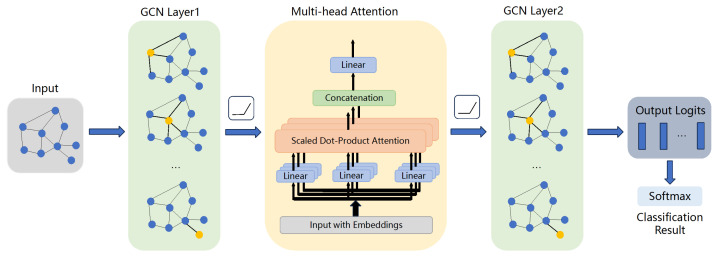
Architecture of the proposed Graph Neural Network (GNN) model.

The specific architecture of our proposed model, which integrates both graph convolution and attention mechanisms, is illustrated in Figure 2. The input node features first pass through a graph convolutional layer, which acts as a powerful feature extractor by aggregating information from each node’s immediate neighborhood. The output from this layer is then fed into a graph attention layer. Unlike a standard Graph Convolutional Network, this layer uses a self-attention mechanism to dynamically assign different importance weights to various nodes within a neighborhood, allowing the model to focus on the most relevant sensor features for the classification task. Finally, the attention-weighted features are passed through a second graph convolutional layer, which transforms the high-dimensional hidden representations into the final output dimension corresponding to the number of operational conditions. A ReLU activation function is applied after the first two layers to introduce nonlinearity, enabling the model to learn more complex relationships.

### 2.3. K-Nearest Neighbor Graph Convolution Network

A Graph Convolutional Network (GCN), as a deep learning model specifically designed for processing graph-structured data, requires an explicit graph structure as its input. However, in practical industrial scenarios, data is typically collected from multidimensional sensor sequences and do not inherently possess a graph structure; explicit physical or logical connections between nodes are often absent. As a result, a GCN cannot be directly applied to such data. To address this issue, this paper proposes the use of the K-Nearest Neighbor (K-NN) algorithm to dynamically construct graph structures based on the similarity of sample features in the feature space. This method infers relational associations between nodes from the data itself, thereby explicitly generating graph representations suitable for GCNs.

The K-Nearest Neighbor (K-NN) algorithm is a classical non-parametric supervised learning method that has been widely adopted in classification and regression tasks [24]. Its fundamental principle can be described as follows: in the feature space, the class or value of a test sample is determined by the majority vote or averaging mechanism applied to its k closest training samples [25]. For a given query sample, K-NN first calculates the distance between this sample and all samples in the training set using a selected distance metric. The k training samples with the smallest distances are selected to form the neighborhood. For classification tasks, a majority voting mechanism is employed, meaning the predicted class of the query sample is assigned as:

(3) $$y_{q}=\arg\max_{c}\sum_{x_{i}\in N_{K}\left(x_{q}\right)}I\left(y_{i}=c\right)$$

Among these, I(·) represents the indicator function, and  denotes the class label.

The key parameters of the K-NN algorithm are the selection of the value of *k* and the choice of the distance metric. An excessively small *k* value may lead to an overly complex model that is susceptible to noise or outliers, resulting in overfitting; conversely, an overly large *k* value may cause underfitting and weaken the model’s ability to capture local patterns in the data. The optimal value of *k* is often selected through cross-validation on a validation set. Additionally, a method for measuring the proximity between data points must be defined. For continuous data, the most commonly used metric is Euclidean distance, which calculates the straight-line distance between two points across all dimensions to quantify similarity. Manhattan distance, which computes the sum of absolute differences along each coordinate axis, is also widely adopted. Both metrics have been demonstrated to perform effectively in data analysis and classification tasks.

For any two sample vectors *i* and *j* from the baseline training set, the Euclidean distance between them is defined by the following equation:

(4) $$d\left(i,j\right)=\sqrt{\sum_{k=1}^{m}{\left(x_{ik}-x_{jk}\right)}^{2}}$$

In this equation, *m* is the feature dimensionality, and $x_{ik}$ and $x_{jk}$ correspond to the k-th feature of samples *i* and *j*, respectively.

The Manhattan distance is used to measure the sum of the absolute differences between two points along each coordinate axis in a linear space. For two points p and q in a D-dimensional space, the Manhattan distance is defined as follows:

(5) $$D\left(p,q\right)=\sum_{k=1}^{D}\left|p_{k}-q_{k}\right|$$

where $p_{k}$ and $q_{k}$ are the k-th coordinates of points p and q, respectively.

Cosine similarity is a method used to measure the angle between two non-zero vectors, with its core principle lying in the assessment of directional consistency. Unlike magnitude-based metrics, it is invariant to the dimensional magnitudes of the vectors, making it particularly suitable for applications where alignment in direction is emphasized over differences in magnitude. For two non-zero vectors A and B, the cosine similarity between them can be calculated using the following formula:

(6) $$\cos\left(A,B\right)=\frac{A\cdot B}{\left|A|\;|B\right|}$$

where A·B denotes the dot product of the vectors, and |A| and |B| represent the magnitudes of vectors A and B, respectively. After a comparative analysis of the three aforementioned metrics, Euclidean distance is adopted as the similarity measurement method in the K-NN model. This choice is motivated by the physical nature of the sensor data; as the features represent continuous physical quantities, the Euclidean distance provides an intuitive and effective measure of the overall similarity between different operational states.

The principle of the K-Nearest Neighbor Graph Convolutional Network (KNN-GCN) method lies in its utilization of the K-Nearest Neighbor (K-NN) algorithm to adaptively mine local spatiotemporal correlations among multi-sensor readings of deep-sea manned submersibles, thereby constructing a topological graph structure that reflects the coupling relationships among equipment states. This structure is subsequently processed by a Graph Convolutional Network (GCN) to achieve feature enhancement and collaborative identification. Specifically, sensor readings collected at different time instances are treated as nodes in the graph. The feature similarity between these nodes is measured using Euclidean distance. In this pager, the distance d(i,j) calculates the similarity between two sensor snapshots *i* and *j* from the submersible. Here, *m* represents the total number of sensors being monitored, and $x_{ik}$ is the specific reading from the k-th sensor for snapshot *i*. A smaller distance d(i,j) indicates that the submersible was in a very similar operational state at the times these two snapshots were recorded. Based on Euclidean distance, feature similarities between nodes are measured to establish local connections for each node via its K-Nearest Neighbors, forming a sparse adjacency matrix. On this basis, the GCN iteratively updates node features through multi-layer neighborhood aggregation, effectively integrating local patterns and global dependencies within the multi-sensor data to achieve robust recognition of operational states or external environmental characteristics of the deep submersible.

The proposed KNN-GCN-based condition identification algorithm transforms non-graph sequential sensor data into a graph structure to leverage GCNs for accurate classification. Conceptually, this process can be broken down into two main stages. First, in the graph construction stage, each multi-dimensional sensor reading at a specific time point is treated as an individual node. The K-NN algorithm is then employed to establish the graph’s structure. For a given node, its Euclidean distance to all other nodes is calculated, and edges are formed by connecting it to its K-Nearest Neighbors. This transforms the entire collection of isolated data points into a meaningful graph where proximity in the feature space is encoded as a direct connection. Second, in the feature aggregation stage, the newly created graph is fed into the GCN. The GCN then iteratively updates each node’s features by aggregating information from its immediate neighbors (as defined by the K-NN step). This allows each data point to learn from its local context, leading to richer, more separable feature representations for the final classification task. The main steps are summarized in Algorithm 2, with a detailed description as follows:

1. Data Input and Preprocessing: The multi-source sensor time-series dataset of the deep submersible is input, denoted as D=(X,Y), where $X\in R^{N\times M}$ represents the data matrix with *N* samples and *M* features, and $Y\in Z^{N}$ is the corresponding operational condition label vector. Data cleaning and outlier removal are performed to eliminate anomalies, resulting in a normalized dataset $\tilde{D}$.
2. Graph Convolutional Network Construction: Based on the processed data matrix $\tilde{X}$, the K-NN algorithm is utilized to dynamically construct local neighborhood relationships for each sample node, thereby generating the graph-structured data. The obtained graph structure $G=(\tilde{X},A)$ is utilized as input to the Graph Convolutional Network (GCN) for model training.
3. Model Update Strategy: When new operational conditions emerge, the aforementioned steps are repeated. Initially, outlier detection is performed on the newly acquired data. Subsequently, a new K-NN model is constructed to compute the neighborhood relationships between nodes. Based on the updated information among nodes, an adjacency matrix for the new data is generated. This process does not recompute the entire graph structure. Instead, the new data points are treated as a distinct set of nodes. A K-NN model is constructed exclusively on this new data to generate a new set of edges, forming a new subgraph. The updated graph for the model is then created by concatenating the feature matrix and edge index of the original graph with those of the new subgraph. This process ensures the continuity of the entire data graph while effectively incorporating features of the new operational conditions.

The model is trained using data that has been augmented with information from the new operational conditions. This step is critical for the model update process. By incorporating new data, the model is enabled to adapt to novel operational scenarios, thereby enhancing its recognition accuracy and robustness under varying conditions. During this process, particular emphasis is placed on the model’s generalization capability to prevent overfitting. Furthermore, regular performance evaluations are conducted to promptly identify and rectify potential biases or deficiencies, ensuring the long-term stable operation of the system.
**Algorithm 2** Graph Convolutional Neural Network based on K-NN**Default values:** k=5, Dropout =0.5, α=0.01, Epoch =100.**Require:** 
Nearest Neighbor *k*, Regularization Drop Rate Dropout, the batch size *m*, Adam hyperparameters α.1:**Data Preparation and Preprocessing**2:**Constructing Graph Data Based on K-NN**3:     initializing the K-Nearest Neighbors Model4:     fit k-nearest neighbors model5:     find the K nearest neighbors for each sample6:     construct an adjacency matrix *A*7:     constructing graph structure data G=(X,A)8:**Train the graph convolutional model using the generated graph data**9:**Processing incremental data**10:     constructing the graph structure of new data11:     merge new and old map data12:**Retrain the graph convolutional model**13:**return** current operating condition

## 3. Experiment Implementation Details

### 3.1. Description of Deep Submersible Monitoring Data

The data utilized in this study consisted of historical records from the ‘Jiaolong’ manned submersible, collected during real-world deep-sea missions. The operational conditions were meticulously labeled based on official mission logs. Initially, two distinct classes of operating conditions were analyzed: ‘descent’ (labeled as class 0) and ‘cruising’ (labeled as class 1). To test the model’s adaptability, a third condition, ‘Robotic Arm Operation’ (labeled as class 2), was subsequently introduced. Due to national security and proprietary restrictions governed by the National Deep Sea Center, further details about the vessel’s specifications cannot be disclosed. The full dataset amounts to a total of 77,449 samples, comprising nine variables. One of these, a timestamp, was removed during preprocessing as it was not relevant for the classification task, leaving eight features for model training and analysis. The data was recorded at a sampling frequency of 4 Hz. The collected raw data was subsequently subjected to an outlier detection process, as detailed below, to remove anomalous entries. In the initial experimental phase, a dataset containing 4004 samples was used, which encompassed two distinct classes of operating conditions. This dataset was partitioned via cross-validation into a training set and a testing set, each containing 2002 samples. As the operational scope expanded, the dataset was augmented with a third class of operating conditions. After outlier removal, this new dataset contained 2038 samples. The specific division of the training and testing sets for both the original (class 2) and new (class 3) scenarios is presented in Table 1.

The submersible operates in a harsh, complex, and variable environment, leading to significant noise and strong signal interference in the sensor data. These issues pose challenges for the direct construction of a robust condition classification model. To address this problem, we propose an outlier detection method that combines a sliding window approach with the Median Absolute Deviation (MAD). The MAD method is an improvement over traditional mean- and standard deviation-based approaches, offering a more robust technique for outlier identification. This method utilizes the sample median and the Median Absolute Deviation—metrics that are less sensitive to outliers—to define the boundaries for anomalous data points. The formulas are as follows:

(7) $$\mathrm{MAD}=\mathrm{median}(|Y_{i}-\mathrm{median}\left(Y_{i}\right)|)$$

(8) Upper=median+3·MAD

(9) Lower=median−3·MAD

**Figure 3:**
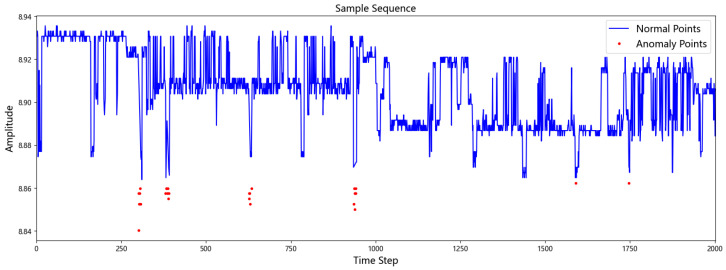
Anomaly point detection.

In these equations, $Y_{i}$ represents the sequence of data points within the current sliding window, media $\left(Y_{i}\right)$ is the median value of that sequence, and MAD is the Median Absolute Deviation. The Upper and Lower values define the thresholds for identifying outliers; any data point outside this range is classified as an anomaly. A window width and a step size are predefined to iteratively extract subsequences from the data. For the data within each window, the median is first calculated. Then, the absolute deviation of each point from this median is computed. Subsequently, the Median Absolute Deviation (MAD) of this sequence of absolute deviations is calculated according to Equation (7). Finally, each data point is classified as an outlier based on the criteria defined in Equations (8) and (9). In this study, this outlier detection method was applied to the 6th variable feature of the dataset. This targeted approach was taken because a preliminary exploratory analysis revealed that this specific variable exhibited a significantly higher frequency of anomalous spikes and noise compared to the other features, which were relatively clean and stable. The identified outliers were removed to enhance the overall quality of the data. The results of the outlier detection are illustrated in Figure 3.

The core methodology of this paper is based on the Graph Convolutional Network (GCN). However, the data is originally collected in a time-series format. To adapt this temporal data for the processing requirements of a GCN, it is necessary to convert it into a graph data structure. This transformation allows us to leverage the advantages of GCNs in processing structured graph data. The data preprocessing pipeline involves the following three main steps:

Step 1: Data Collection and Initial Processing. Raw time-series data is collected from sensors located on various equipment units of the submersible. This data is then cleaned using the aforementioned sliding window with the MAD method to remove outliers, thereby ensuring data quality and integrity.

Step 2: Graph Construction. A non-parametric learning method, the K-Nearest Neighbor (K-NN) algorithm, is employed to identify the nearest neighbors for each node in the dataset. The connectivity relationships between each node and its nearest neighbors are then calculated to construct an adjacency matrix.

Step 3: Dataset Partitioning. The transformed graph data is partitioned into training and testing sets to support the model’s training, tuning, and evaluation processes.

### 3.2. Hyperparameter Tuning

Initially, the K-Nearest Neighbor (K-NN) algorithm is employed for data transformation. The K-NN algorithm constructs the graph by first identifying the K nearest neighbors for each node based on Euclidean distance. Based on empirical evaluation, K is set to 5 for optimal performance. Edges are then established between each node and its five identified neighbors. This iterative process across all nodes results in a graph structure, which is then encoded into a sparse adjacency matrix and a graph data object for input into the GCN. In the adjacency matrix, the corresponding element is assigned a value of 1 if two nodes are neighbors (or weighted according to their distance), and 0 otherwise. Following the data preprocessing, a Graph Convolutional Network (GCN) is utilized to perform the identification of operating conditions. In graph-structured data, the features and labels of a node are largely influenced by its neighbors. Consequently, this paper adopts a two-layer GCN architecture, which can efficiently capture and learn local patterns of node features by aggregating information from each node and its immediate vicinity. The first graph convolutional layer processes the input features, and the ReLU activation function is applied to introduce nonlinearity. This is followed by a Dropout layer with a rate of 0.5 for regularization. Finally, the features are passed through a second graph convolutional layer and normalized via a Softmax layer to yield the class probability distribution. The model is trained using the Adam optimizer with a learning rate of 0.01 for 100 epochs and with a batch size of 400.

### 3.3. Experimental Design, Comparative Algorithms, and Evaluation Methodology

To validate the superiority of the proposed K-Nearest Neighbor Graph Convolutional Model (KNN-GCM) in identifying the operational conditions of deep-sea manned submersibles, historical data containing two types of operational conditions were first used to train the model. Subsequently, new operational condition data were introduced to verify that the proposed method could adaptively expand the historical operational condition dataset and effectively identify unknown conditions. Furthermore, to objectively evaluate the performance of the proposed approach and further demonstrate the accuracy of the K-Nearest Neighbor Graph Convolutional Network in recognizing operational conditions under dynamic scenarios, three traditional algorithms were selected for comparative experiments. These baseline models were chosen to represent the broad class of methods that process multi-sensor data as a collection of independent features or channels. This category conceptually includes not only traditional algorithms like Random Forest but also advanced time-series classifiers such as 1D-CNNs and Transformers. Although these deep learning models excel at capturing intricate temporal dependencies within each channel, they do not inherently model the explicit, graph-like spatial correlations between different sensors—a core focus of this paper. Consequently, the selected baselines are sufficient to validate our central hypothesis: for tightly coupled systems like submersibles, the paradigm of explicitly modeling inter-sensor relationships is critical for robust performance. The selected algorithms are as follows:

1. Decision Tree [26]. This method constructs a tree-like structure based on feature threshold splitting to make decisions. The model is intuitive and comprehensible, and capable of automatically handling nonlinear relationships. However, it is prone to overfitting.
2. Random Forest [27]. As an ensemble learning algorithm, it constructs multiple decision trees and aggregates their voting results for prediction. It effectively mitigates overfitting, enhances generalization capability and robustness, and performs well with high-dimensional data.
3. Logistic Regression [28]. As a generalized linear model, it employs the Sigmoid function to map linear combinations into probability outputs. It is suitable for binary classification problems and offers strong interpretability. However, its ability to capture complex nonlinear patterns among features is limited.

To more intuitively demonstrate the performance of the proposed K-Nearest Neighbor Graph Convolutional Model, a confusion matrix was adopted as a key evaluation metric. The confusion matrix is an intuitive and highly informative table used in machine learning to assess the performance of classification models. It goes beyond providing a single accuracy value by offering a detailed comparison between the model’s predictions and the actual ground truth labels. For multi-class classification problems, the confusion matrix is expanded into an N×N matrix, where *N* denotes the number of classes. The rows represent the true classes, while the columns represent the predicted classes. By examining the diagonal entries, which indicate the number of correctly predicted samples, the classification accuracy of the model can be precisely calculated.

## 4. Results and Discussion

### 4.1. Model Performance Evaluation

To validate the effectiveness of the proposed model, training and testing were first conducted using the original operational condition data. To ensure a fair and unbiased evaluation, all performance metrics and results presented hereafter are reported exclusively on the test set. Subsequently, to simulate potential condition variations in real-world operations, a new operational condition category was introduced into the dataset. The model was then updated and retrained by integrating the adjacency matrix.

**Figure 4:**
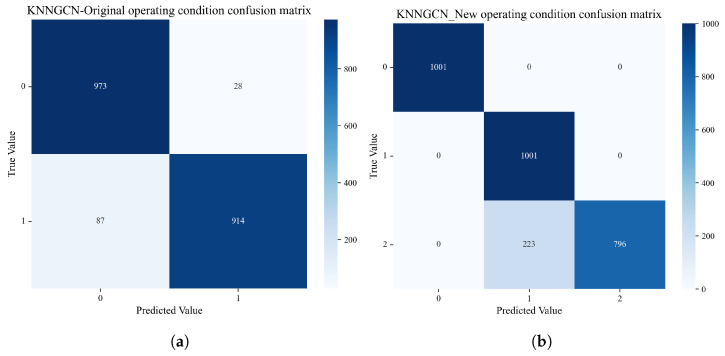
Confusion matrix of KNN-GCN under varying conditions. (**a**) Confusion matrix on the original two-class dataset. (**b**) Confusion matrix on the new three-class dataset after introducing an additional operating condition.

Figure 4 displays the confusion matrices of the classification results obtained by the KNN-GCN model under both the original and newly added operational conditions. In the original two-condition classification task, the model demonstrated exceptional performance. Specifically, all test samples belonging to “Condition 0” were predicted with 100% accuracy, while the prediction accuracy for “Condition 1” reached 93.4% on the test set. When a new “Condition 2” was introduced to simulate changing operational conditions, the model rapidly adapted to the new data distribution through an effective update strategy. As observed in the confusion matrix, the model maintained 100% recognition accuracy for both “Condition 0” and “Condition 1”, while also achieving a prediction accuracy of 79.6% for the newly introduced “Condition 2”.

These results fully demonstrate that the proposed model not only exhibits excellent feature extraction and classification capabilities but also shows strong adaptability and robustness when confronted with changing operational conditions. The introduction of the graph structure provides rich structural information for time-series data analysis, confirming the effectiveness and reliability of the proposed method in variable-condition recognition tasks.

### 4.2. Comparative Experiments and Analysis

To further demonstrate the advantages of the proposed KNN-GCN model and validate its accuracy in dynamic operational condition identification, three benchmark models—Decision Tree (DT), Logistic Regression (LR), and Random Forest (RF)—were included for comparative experiments under the same experimental settings.

#### 4.2.1. Performance Comparison Under Original Operational Conditions

**Figure 5:**
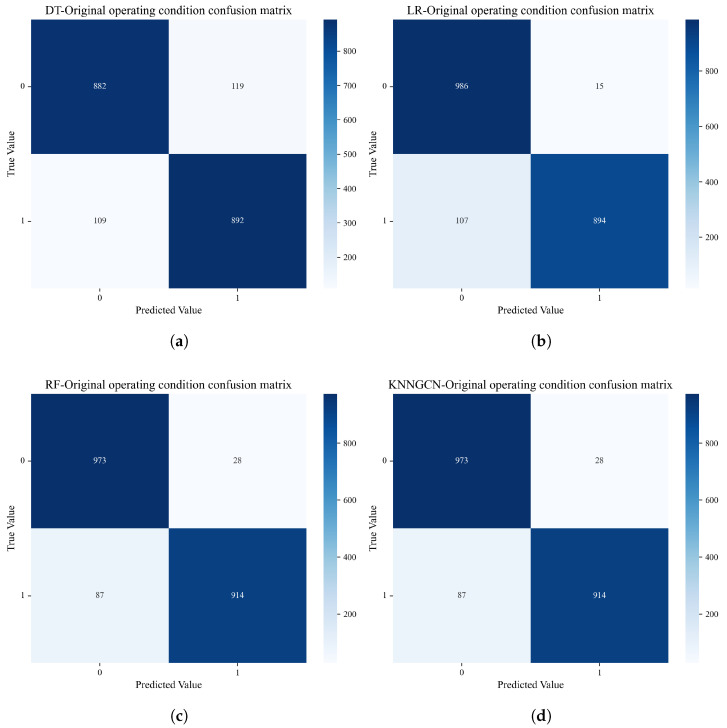
Comparison of confusion matrices on the original two-class dataset. This figure compares the performance of four different models on the original operating conditions: (**a**) Decision Tree (DT); (**b**) Logistic Regression (LR); (**c**) Random Forest (RF); (**d**) the proposed KNN-GCN model.

Figure 5 presents the classification confusion matrices of the four models set under the original operational conditions (two-class scenario). Table 2 quantitatively summarizes the classification accuracy of each model. The results demonstrate that the KNN-GCN model achieved the highest operational condition identification accuracy of 96.65%, significantly outperforming Decision Tree (88.61%), Logistic Regression (92.66%), and Random Forest (94.26%). This superior performance indicates that by transforming time-series data into a graph structure, the model can more effectively capture the underlying complex interdependencies among various variables (nodes). The rich topological information provided by the graph data enables the model to fully leverage the coupling effects between sensor data, thereby surpassing traditional machine learning models in both feature extraction and classification capability.

#### 4.2.2. Performance Comparison Under Newly Introduced Operational Conditions

**Figure 6:**
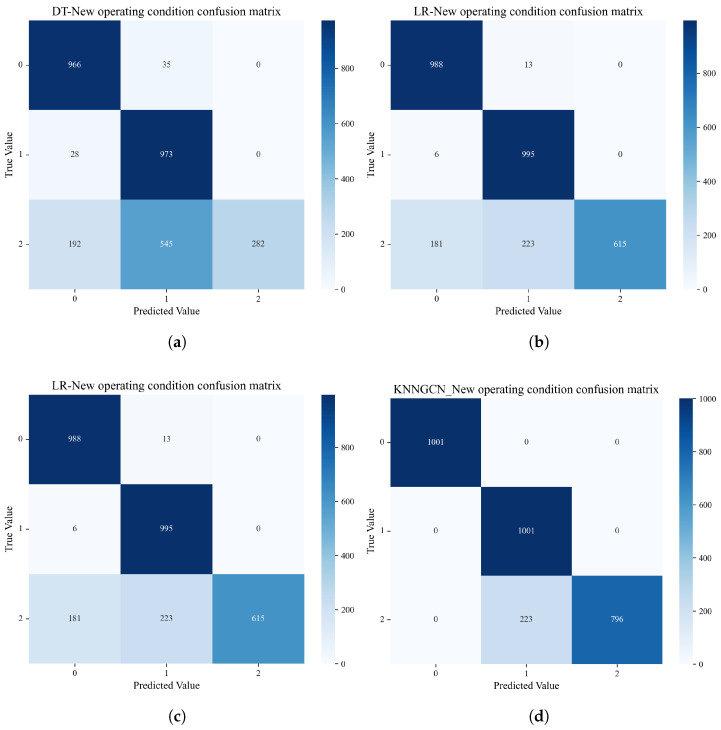
Comparison of confusion matrices on the new three-class dataset. This figure compares the performance of the four models when a new operating condition is introduced: (**a**) Decision Tree (DT); (**b**) Logistic Regression (LR); (**c**) Random Forest (RF); (**d**) the proposed KNN-GCN model.

Figure 6 displays the classification confusion matrices of the four models after the introduction of the new operational condition (three-class scenario). As clearly observed from the figure, when the number of operational conditions increases, the Decision Tree, Logistic Regression, and Random Forest models all demonstrate unsatisfactory performance in classifying the new condition, with their overall accuracy experiencing a significant degradation. In contrast, owing to its effective update strategy, the KNN-GCN model maintains strong adaptability to the new operational condition. As summarized in Table 2, it achieves a total accuracy of 92.62% on the test set.

**Figure 7:**
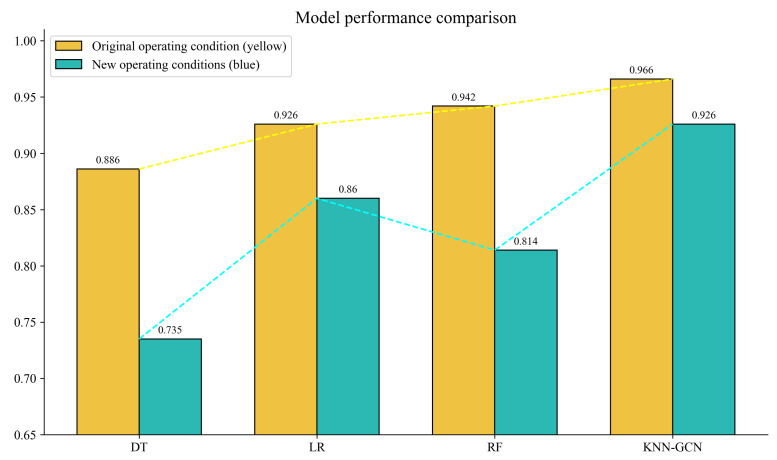
Model performance comparison histogram.

As further corroborated by the performance comparison histogram shown in Figure 7, although the accuracy of the KNN-GCN model experienced a slight decrease compared to its performance under original conditions due to increased operational complexity, it still significantly outperformed all other comparative models. This result strongly demonstrated the robustness and forward-looking capability of the proposed method in handling dynamically changing operational conditions. By integrating graph structure and neighborhood features, the model effectively updated itself to adapt to new classification tasks.

### 4.3. Interpretability Analysis of the Model

Graph Convolutional Networks (GCNs) are often regarded as “black-box” models due to their highly complex architecture and nonlinear information propagation mechanisms, which result in a lack of transparency in their internal decision-making processes. To ensure the rationality, reliability, and domain knowledge consistency of model decisions, there is a pressing need to introduce interpretability methods. For this purpose, this paper employs the SHAP (SHapley Additive exPlanations) value-based approach to provide visual explanations for the model.

The SHAP method quantifies feature importance by calculating the Shapley value of each feature’s contribution to an individual prediction [29]. Its core procedure consists of the following steps:

1. Enumerating all possible feature subsets (combinations).
2. For each feature, computing its marginal contribution when added to different subsets.
3. Taking a weighted average of all marginal contributions to obtain the final SHAP value for the feature.

**Figure 8:**
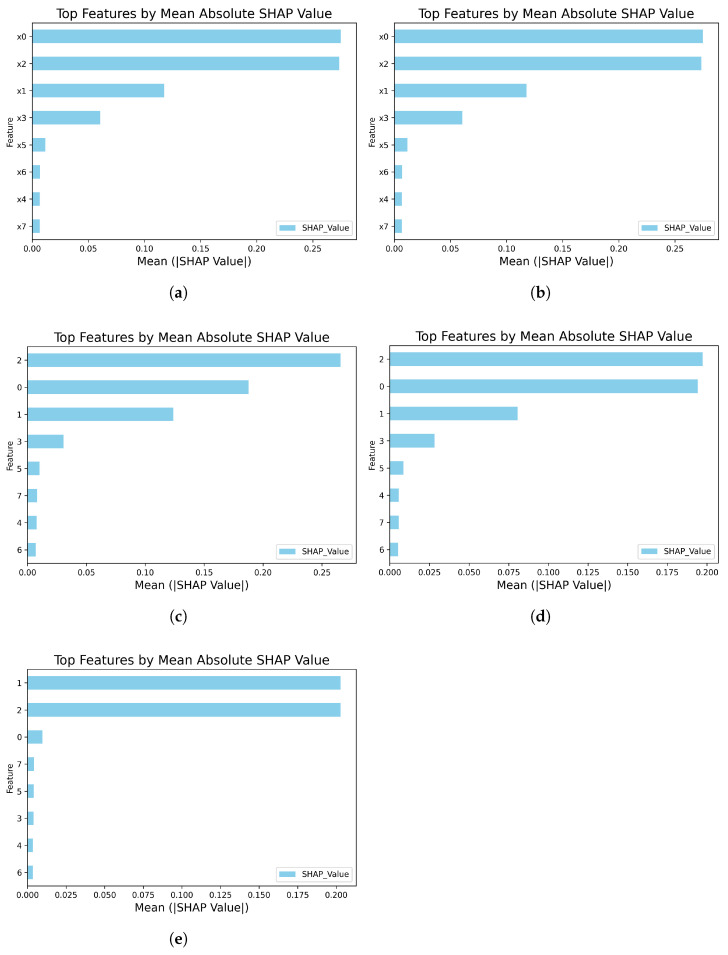
Global feature importance plot. The figure illustrates the top features ranked by their mean absolute SHAP values, indicating global feature importance for different class predictions: (**a**) Feature importance for class 0 under original conditions. (**b**) Feature importance for class 1 under original conditions. (**c**) Feature importance for class 0 with new condition data. (**d**) Feature importance for class 1 with new condition data. (**e**) Feature importance for the new class 2.

**Figure 9:**
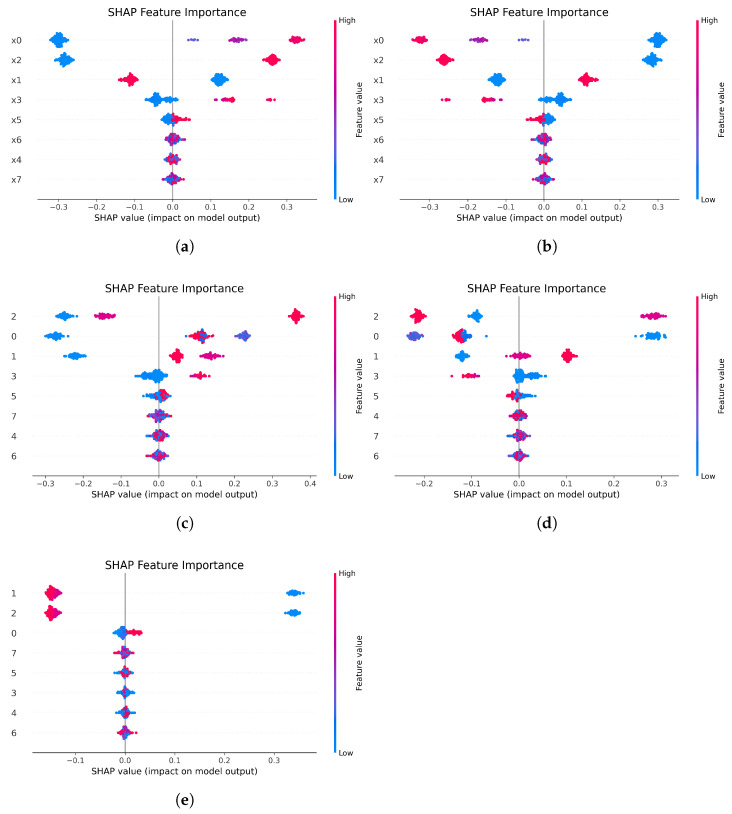
SHAP beeswarm plot. This figure provides a detailed summary of SHAP values for each feature across all samples, revealing both the magnitude and direction of their impact on the model’s output for each class: (**a**) SHAP values for class 0 under original conditions. (**b**) SHAP values for class 1 under original conditions. (**c**) SHAP values for class 0 with new condition data. (**d**) SHAP values for class 1 with new condition data. (**e**) SHAP values for the new class 2.

Figure 8 and Figure 9 present the global feature importance ranking and the SHAP beeswarm plot, respectively. Among them, Figure 8a,b correspond to the analysis results of class 0 and class 1 under the original operational conditions, while Figure 8c–e correspond to the analysis of class 0, 1, and 2 under the newly added conditions. In the beeswarm plot, each point represents a data sample. Its position along the horizontal axis indicates the SHAP value of the feature for that sample’s prediction (positive values denote positive contributions, negative values denote negative contributions). The color of the point represents the magnitude of the feature value (typically, red indicates high values and blue indicates low values).

Beyond a superficial ranking of feature importance, a deeper analysis of the SHAP values reveals that the model has learned to identify physically meaningful patterns that align with the submersible’s core operational principles. For instance, in classifying the ‘Cruising’ condition (class 1), the model correctly identifies primary propulsion and power consumption sensors (x0, x1) as the most salient discriminators. The SHAP plots (Figure 8d and Figure 9d) show that high values for these features strongly and positively influence the prediction, a finding that is mechanistically sound given that sustained cruising requires significant and continuous power output. Conversely, when identifying ‘Robotic Arm Operation’ (class 2), the model’s focus shifts dramatically to a key hydraulic system indicator (x2). High values for this feature, as seen in Figure 8e and Figure 9e, provide a strong positive push towards this classification, directly reflecting the physical reality that robotic arm engagement is a hydraulically intensive task. This ability to dynamically shift focus based on the operational context demonstrates that the model’s internal logic is consistent with established engineering knowledge. Such alignment between data-driven findings and domain expertise moves beyond simple interpretability to establish a higher degree of trustworthiness in the proposed method.

Through SHAP analysis, not only has the classification performance of the model been validated, but its internal decision-making mechanism has also been thoroughly revealed, significantly enhancing the model’s credibility and interpretability.

## 5. Conclusions

Focusing on the challenges of dynamic condition variations in deep-sea manned submersibles operating in complex underwater environments, strong coupling among multi-sensor data, and the limited identification capability of traditional methods, this paper proposes an online condition identification model based on a K-Nearest Neighbor Graph Convolutional Network (KNN-GCN). By converting time-series sensor data into graph structures and adaptively constructing node neighborhood relationships using the K-NN algorithm, the model effectively captures implicit spatiotemporal coupling characteristics and global dependencies within the data. Experimental analyses based on deep submersible operational data demonstrate that the proposed method achieves high identification accuracy prior to changes in operational conditions. Even when new conditions emerge, the method continues to rapidly and efficiently identify the current condition type online, highlighting its strong feature extraction capability and condition adaptation performance. Furthermore, an interpretability analysis approach based on SHAP values is introduced to reveal the contribution levels of different sensor variables to classification decisions, thereby enhancing the model’s credibility and comprehensibility. This provides a reliable algorithmic foundation for intelligent monitoring and fault diagnosis of deep-sea manned submersibles. Future work will focus on lightweight model design and real-time performance optimization to meet the deployment requirements of embedded platforms on deep-sea manned submersibles, as well as exploring their application potential in more complex scenarios such as multimodal and cross-condition settings.

## Figures and Tables

**Table 1:** Dataset division.

Original Operating Condition			New Operating Condition		
(Two Types of Conditions)			(Three Types of Conditions)		
Sample Size Train/Test	Type	Label	Sample Size Train/Test	Type	Label
1001/1001	type1	0	1001/1001	type1	0
1001/1001	type2	1	1001/1001	type2	1
			1019/1019	type3	2

**Table 2:** Algorithm comparison.

Model	Original Operating Condition (Two Types of Conditions)	New Operating Condition (Three Types of Conditions)
DT	0.8861	0.7352
LR	0.9266	0.8600
RF	0.9426	0.8143
KNN-GCN	*0.9665*	*0.9262*

## Data Availability

The data presented in this study is available on request from the corresponding author due to national security and proprietary restrictions concerning operational data from the ‘Jiaolong’ manned deep-sea submersible, which is governed by the National Deep Sea Center.
